# Associations of circulating apolipoprotein J and myostatin with sarcopenia in older adults with and without type 2 diabetes: a cross-sectional study

**DOI:** 10.3389/fendo.2025.1592112

**Published:** 2025-06-30

**Authors:** Inha Jung, Da-Hye Shin, Hyun Joo Cho, So Young Park, Da Young Lee, Ji Hee Yu, Nan Hee Kim, Minjin Lee, Young-Bum Kim, Ji A Seo

**Affiliations:** ^1^ Division of Endocrinology and Metabolism, Department of Internal Medicine, Korea University College of Medicine, Seoul, Republic of Korea; ^2^ Division of Endocrinology, Diabetes and Metabolism, Department of Medicine, Beth Israel Deaconess Medical Center, Harvard Medical School, Boston, MA, United States

**Keywords:** insulin resistance (IR), diabetes mellitus (DM), apolipoprotein J (ApoJ), myostatin (MSTN), appendicular skeletal muscle mass (ASM), handgrip strength (HS), physical performance (PP)

## Abstract

**Introduction:**

Type 2 diabetes mellitus (DM) is a known risk factor for sarcopenia. Apolipoprotein J (ApoJ) and myostatin (MSTN) have been implicated in muscle glucose metabolism. We aimed to examine the association between serum ApoJ and MSTN levels and sarcopenia in older adults, with and without DM.

**Methods:**

This cross-sectional study included 130 community-dwelling adults aged 65–92 years. Serum ApoJ and MSTN levels were measured using ELISA. Sarcopenia was defined as low appendicular skeletal muscle index (ASMI) with low handgrip strength (HS) and/or poor physical performance (PP). Associations were analyzed using age- and sex-adjusted models and logistic regression.

**Results:**

Sarcopenia was present in 17.7% of participants. Those with sarcopenia had higher ApoJ levels than those without (p = 0.022). ApoJ levels did not differ by DM status, but MSTN levels were lower in participants with DM (p = 0.012). MSTN levels were positively associated with ASMI, HS, and PP. In logistic regression, ApoJ was independently associated with sarcopenia (OR = 1.027, p = 0.006) and severe sarcopenia (OR = 1.041, p = 0.027), while MSTN was inversely associated with severe sarcopenia (OR = 0.980, p = 0.025). The highest sarcopenia prevalence (26.7%) was observed in the high ApoJ/low MSTN group, and the lowest (0%) in the low ApoJ/high MSTN group.

**Discussion:**

Elevated ApoJ and reduced MSTN levels are associated with sarcopenia in older adults. These biomarkers may play opposing roles and serve as potential predictors for sarcopenia.

## 1 Introduction

Sarcopenia, characterized by the progressive and generalized loss of skeletal muscle mass and strength, has emerged as an important medical condition owing to its association with various adverse health outcomes, including increased risk of falls, functional decline, and higher mortality rates (1, 2). The prevalence of sarcopenia among individuals aged 60 years and older ranges from 10% to 27%, depending on the definition and diagnostic criteria used, established by the European Working Group on Sarcopenia in Older People (EWGSOP) or the Asian Working Group for Sarcopenia (AWGS) (3, 4). Both the EWGSOP and AWGS incorporate muscle mass, muscle strength, and physical performance as the core components of their diagnostic criteria for sarcopenia. However, the EWGSOP considers low muscle strength as the primary indicator to initiate further evaluation, whereas the AWGS requires low muscle mass as an essential condition for diagnosis (5, 6). With the ongoing global aging trend, the burden of sarcopenia is anticipated to increase significantly, highlighting the urgent need for reliable diagnostic biomarkers and effective therapeutic targets. However, despite extensive research, no universally accepted biomarker for sarcopenia has yet been identified.

Recent studies have shown a higher prevalence of sarcopenia in individuals with type 2 diabetes mellitus (DM) compared to those without DM (7, 8), and accumulating evidence suggests a bidirectional relationship between DM and sarcopenia (9, 10). Shared pathophysiological mechanisms include insulin resistance (IR), chronic inflammation, and mitochondrial dysfunction (11, 12). Skeletal muscle plays a pivotal role in glucose metabolism and IR while also functioning as an endocrine organ that secretes myokines, which mediate inter-organ crosstalk and metabolic homeostasis (13–16).

Among the various endocrine factors involved in muscle metabolism, myokines and hepatokines have received growing attention. Hepatokines including apolipoprotein J (ApoJ), also known as clusterin, have been recognized as important modulators of muscle metabolism. ApoJ is secreted by the liver and transported to skeletal muscle, where it modulates glucose metabolism and insulin signaling (17, 18). *In vitro* studies suggest that ApoJ may influence myoblast viability and promote muscle cell senescence under metabolic stress (19, 20). However, it remains unclear whether circulating ApoJ levels in humans are associated with muscle mass or function, and whether such associations differ depending on metabolic conditions such as the presence of DM.

Myostatin (MSTN), the first recognized myokine (21), has been studied in preclinical models. As a regulator of muscle growth, MSTN inhibition in animal models has been shown to enhance muscle mass and improve metabolic parameters (22, 23). These findings have led to the development of MSTN inhibitors as potential therapeutic agents for sarcopenia. However, clinical trials in humans have produced inconsistent results, likely due to the significantly lower circulating MSTN levels in humans compared to animals and the lack of comprehensive understanding of its expression and regulatory mechanisms within the human body (24, 25). Furthermore, human clinical trials have failed to demonstrate consistent functional improvements with MSTN inhibition (26, 27), highlighting the need for further investigation as increased muscle mass does not necessarily result in improved muscle function.

Therefore, we aimed to examine whether circulating ApoJ and MSTN levels are associated with sarcopenia in older adults, focusing on key diagnostic domains such as muscle mass, handgrip strength, and physical performance (PP). To further explore the potential metabolic influence, we also compared sarcopenia-related parameters and circulating ApoJ and MSTN levels between individuals with and without DM.

## 2 Materials and methods

### 2.1 Study design and population

This cross-sectional study recruited community-dwelling participants aged 65 years or older who visited the Korea University Ansan Hospital. The participants were stratified into four groups based on sex and DM status, with each group comprising 30–40 individuals. Individuals who had lost the ability to provide informed consent due to mental disorders such as dementia, were unable to stand or walk independently, had a history of malignant tumors within the past 5 years, or experienced cardiovascular or cerebrovascular events requiring hospitalization within the past 6 months were excluded. Furthermore, individuals who used systemic steroids within the past month, with a glycated hemoglobin (HbA1c) level of >10% or a change in HbA1c levels of more than 2% within 3 months, whose diabetes medications were changed within the past month, with a history of hospitalization for more than 3 days within the past month, with a weight change of more than 10% within the past 3 months, with other conditions or who used medications that may cause significant changes in weight or body composition, or with type 1 DM were also excluded. A total of 133 participants were initially recruited. Among them, two individuals withdrew informed consent during the enrollment process, and one individual was excluded due to missing serum ApoJ measurements. Therefore, a total of 130 participants were ultimately included in the final analysis. This study was exploratory in nature, aiming to investigate the associations between circulating biomarkers and sarcopenia-related parameters, including muscle mass, handgrip strength, and physical performance, in older adults with and without type 2 DM. This study was performed in accordance with the principles of the Declaration of Helsinki of the World Medical Association and was approved by the Institutional Review Board (IRB) of Korea University Ansan Hospital (IRB no. 2022AS0172).

### 2.2 Demographic, anthropometric, and laboratory measurements

All participants completed an interviewer-administered questionnaire used to assess their physical activity level and nutritional status and underwent comprehensive physical examinations. Smoking status was classified as never smoker, former smoker, or current smoker. Physical activity was assessed using the Korean version of the Global Physical Activity Questionnaire (K-GPAQ) (28). Nutritional intake was evaluated through face-to-face consultations with a clinical dietitian using a food frequency questionnaire (29). The body mass index (BMI) was calculated as weight in kilograms divided by height in meters squared. The fasting plasma glucose (FPG), glycated hemoglobin (HbA1c), serum total cholesterol, triglyceride, high-density lipoprotein (HDL) cholesterol, and low-density lipoprotein (LDL) cholesterol levels were measured after a 12-hour overnight fast using an autoanalyzer (ADVIA 1650, Siemens, Tarrytown, NY, USA). High-sensitivity C-reactive protein (hsCRP) levels were measured using an immunoassay (ADVIA1800, Siemens, USA). Serum insulin levels were measured using an immunoradiometric assay kit (INS-IRMA Kit; BioSource, Nivelles, Belgium) and a Packard counter system. IR was estimated using the homeostasis model assessment of IR (HOMA-IR) index with the following formula: HOMA-IR = [fasting insulin (µU/ml) × FPG (mg/dL)]/405 (30). The HOMA of β-cell function (HOMA-β) was calculated using the following formula: HOMA-β = 360 × fasting insulin (µU/ml)/[FPG (mg/dl) – 63] (30). DM was defined as an FBG level of ≥126 mg/dL, an HbA1c level of ≥6.5%, or the current use of diabetes medication.

### 2.3 Measurement of MSTN and ApoJ

Serum levels of MSTN (pg/ml) were measured using the Human Myostatin Quantikine ELISA Kit (R&D Systems, Inc., Minneapolis, Minnesota, USA, Catalog # DGDF80) according to the manufacturer’s protocol (31) Serum levels of ApoJ (ug/ml) were measured using the Human Clusterin Quantikine ELISA Kit (R&D Systems, Inc., Minneapolis, Minnesota, Catalog # DCLU00) following the manufacturer’s protocol. Each sample was tested in duplicate, and the average values were calculated.

### 2.4 Definition of sarcopenia

We evaluated three parameters based on the clinical guidelines of the Korean Working Group on Sarcopenia: appendicular skeletal muscle mass index (ASMI), muscle strength represented by handgrip strength (HS), and physical performance (PP) (32). Sarcopenia was defined as decreased ASMI combined with either low muscle strength or poor PP. Meanwhile, severe sarcopenia was defined as decreased ASMI, low muscle strength, and poor PP.

Appendicular skeletal muscle mass (ASM) was measured as the sum of the lean muscle mass in the upper and lower extremities using dual-energy X-ray absorptiometry (Lunar Radiation, Madison, WI, USA) and was divided by height in meters squared to calculate the ASMI (kg/m^2^). The cutoff values of low ASMI were <7.0 kg/m^2^ for men and <5.4 kg/m^2^ for women (6, 32). HS was measured using a digital dynamometer (TKK-5401; Takei^®^ dynamometer, Tokyo, Japan). The participants were instructed to stand up, fully extend both arms to prevent flexion, and perform two maximum-effort grips. The highest value was recorded as HS (kg). Low muscle strength was defined as an HS of <28 kg for men and <18 kg for women (6, 32).

PP was assessed using the Short Physical Performance Battery (SPPB) (33) and a multi-sensor-based kiosk (AndanteFit, Dyphi, Daejeon, South Korea). The validation process and detailed information about AndanteFit are described in the literature (34). The SPPB comprises three domains: balance, 4-meter gait speed, and 5-time chair stand-up test. Each domain is scored from 0 to 4 points, with a total score of 12 points. In addition to the SPPB score, the time taken to complete the 5-time chair stand test, the timed up and go (TUG) test, and the 4-meter gait speed was measured separately to assess PP. Individuals with an SPPB score of ≤9 were considered as having poor PP. Participants who took more than 10 seconds to complete the 5-time chair stand-up test, had a 4-meter gait speed of less than 1.0 m/sec, or completed the TUG test for more than 12 seconds were considered to have poor PP (32). Further methodological details regarding physical performance assessments and related equipment are available in the **Supplementary Methods**.

### 2.5 Statistical analysis

The baseline characteristics were expressed as numbers (%), means ± standard deviations, or median values (interquartile range [IQR]). Normally distributed continuous variables were analyzed using the independent t-test, while non-normally distributed continuous variables were compared using the Wilcoxon rank-sum test. Categorical variables were assessed using the chi-square test. Non-normally distributed variables, such as aspartate and alanine transaminase, fasting insulin, HOMA-IR, and HOMA-β levels, were expressed as median values with the corresponding IQRs for each group, and these skewed variables were analyzed after logarithmic transformation. The log-transformed values of the measured levels of ApoJ and MSTN approximated a normal distribution. Therefore, the back-transformed mean of the log-transformed values, referred to as the geometric mean, was employed as the primary statistical measure to assess ApoJ and MSTN.

We analyzed covariance (ANCOVA) to determine the differences in the levels of ApoJ and MSTN according to the presence of sarcopenia-related parameters or DM after adjusting for age and sex. Partial Spearman’s correlation analysis was conducted to assess the relationship between ApoJ and MSTN and various clinical variables after adjusting for age and sex. For muscle function parameters, partial Spearman’s correlation analyses were performed within subgroups stratified by DM status, with further adjustments for muscle mass and physical activity. To assess how the effect of sarcopenia-related parameters on ApoJ and MSTN varied by DM status, we performed a multiple linear regression analysis that included an interaction term for sarcopenia-related parameters and DM status while adjusting for sex, age, ASMI, and GPAQ.

Logistic regression models were used to investigate the independent associations between ApoJ, MSTN, and sarcopenia. The analyses were adjusted for confounding variables such as age, sex, BMI, comorbidity, and levels of physical activity measured using the GPAQ. Interaction terms were incorporated to assess how the relationship between ApoJ and MSTN in patients with sarcopenia may vary depending on DM status. Based on the median values of ApoJ and MSTN, we established four groups categorized according to the levels of these biomarkers. The prevalence of sarcopenia was compared between the groups. Fisher’s Exact Test was applied to compare the incidence of sarcopenia and severe sarcopenia across these four ApoJ/MSTN subgroups. Given the small number of events in the sarcopenia and severe sarcopenia groups, we additionally conducted sensitivity analyses using Firth’s penalized maximum likelihood estimation to reduce small-sample bias in logistic regression.

All p values were two-tailed, and a p value of <0.05 was considered significant. Statistical analyses were performed using SAS version 9.4 (SAS Institute Inc., Cary, NC, USA).

## 3 Results

### 3.1 Clinical characteristics of the study participants

**Table 1** shows the clinical characteristics of 130 participants, comprising 66 individuals in the non-DM group and 64 in the DM group). Age, sex, and BMI were similar between the DM and non-DM groups. The HOMA-β index was significantly lower in the DM group, whereas the HOMA-IR and fasting insulin levels showed no significant differences between the two groups. Hypertension and dyslipidemia were more prevalent in the DM group than in the non-DM group. However, systolic blood pressure, total cholesterol, and LDL cholesterol levels were lower in the DM group, likely due to the higher proportion of individuals in this group taking medications for hypertension and dyslipidemia. No significant differences were found in the nutritional intake between the two groups, although the non-DM group exhibited a higher level of physical activity. No difference was found in the age- and sex-adjusted serum ApoJ levels between the DM and non-DM groups [68.0 (62.2–74.3) μg/ml in the non-DM group vs. 70.5 (64.4–77.2) μg/ml in the DM group, p=0.567). In contrast, the age- and sex-adjusted serum MSTN levels were significantly lower in the DM group compared with that in the non-DM group [216.1 (196.0–238.3) pg/ml in the non-DM group vs. 180.3 (163.2–199.1) pg/ml in the DM group, p=0.012). The prevalence of sarcopenia and severe sarcopenia was numerically higher in the DM group than in the non-DM group (21.9% vs. 13.6% and 15.6% vs. 4.6%, respectively); however, a significant difference between the two groups was observed only for severe sarcopenia. Further details on laboratory values, diabetes duration, and sarcopenia-related functional measures are summarized in **Supplementary Table S1**.

**Table 1:** Characteristics of the study participants.

Variables	Non-DM		DM		P-value
N	66		64		
Age* (yr)	75	69−78	74	69−77	0.597
Sex, male (n,%)	32	48.5	34	53.1	0.172
Body mass index, BMI * (kg/m^2^)	24.6	22.7−26.1	24.8	22.6−27.4	0.178
Waist circumference* (cm)	87.0	82−91	91.8	85.3−99	0.001
Systolic blood pressure (mmHg)	135.2	15.0	126.6	14.5	0.001
HbA1c* (%)	5.7	5.5−6.0	6.8	6.5−7.4	<.0001
Fasting plasma glucose* (mg/dL)	100.0	93−107	124.5	110−140	<.0001
Fasting insulin* (μU/ml)	7.2	5.3−12.8	8.3	5.4 −12	0.953
HOMA-IR*	1.9	1.3−3.1	2.5	1.5−3.6	0.140
HOMA-β*	87.3	45.4	56.8	37.8	<.0001
ALT* (IU/L)	17	13−25	17.5	13−26	0.605
AST* (IU/L)	22	19−28	20.5	17.5−27	0.303
CPK* (IU/L)	89.5	68−133	84	64–107.5	0.308
BUN* (mg/dL)	15.1	12.8−19	16.4	12.8–18.8	0.677
Serum creatinine * (mg/dL)	0.79	0.69–0.95	0.87	0.75–1.04	0.062
Total calorie intake* (kcal)	1757.5	1409.9–1963.7	1777.7	1564.2–2015.7	0.458
Protein (g)	63.5	19.3	65.8	17.6	0.475
Carbohydrates (g)	260.4	62.4	268.3	61.0	0.470
Fat (g)	47.10	31.95–59.36	46.10	35.29–55.29	0.725
GPAQ *(METs/week)	1800	1200−3120	1290	550−2795	0.045
Current smoker (n,%)	4	6.1	8	12.5	0.205
Hypertension (n,%)	32	48.5	44	68.8	0.019
Dyslipidemia (n,%)	34	51.5	56	87.5	<.0001
CVD (n,%)	10	15.2	24	37.5	0.004
Apolipoprotein J (μg/ml) *	68.0	62.2−74.3	70.5	64.4–77.2	0.567
MSTN (pg/ml) *	216.1	196.0–238.3	180.3	163.2–199.1	0.012
Sarcopenia (n, %)	9	13.6	14	21.9	0.219
Severe sarcopenia (n, %)	3	4.6	10	15.6	0.035

ALT, alanine aminotransferase; AST, aspartate aminotransferase; BMI, body mass index; BUN, blood urea nitrogen; CPK, creatinine phosphokinase; CVD, cardiovascular disease; DM, diabetes mellitus; GPAQ, global physical activity questionnaire; HbA1c, hemoglobin A1c; HOMA-IR, homeostasis model assessment of insulin resistance; HOMA-β, homeostasis model assessment of β-cell function; METs, metabolic equivalents; MSTN, myostatin.

* Variables that did not meet the assumption of normality, as assessed by the Shapiro–Wilk test and Q–Q plot, are presented as median (interquartile range). These include age, BMI, waist circumference, HbA1c, fasting plasma glucose, ALT, AST, fasting insulin, HOMA-IR and HOMA-β. Skewed variables were log-transformed for statistical analysis where applicable.

Apolipoprotein J (ApoJ) and myostatin (MSTN) levels are expressed as age- and sex-adjusted geometric means.

### 3.2 Comparison of muscle mass and physical performance by DM status

After adjusting for age and sex, participants with DM had lower HS, lower SPPB total score, slower 4-meter gait speed, and longer completion time for 5-time chair stand-up and TUG tests compared with the participants without DM (**Table 2**). In contrast, ASMI, body fat percentage, and thigh circumference did not differ by DM status. Calf circumference was marginally lower in the DM group (p=0.058). These results suggest that the muscle function decline was more pronounced than the muscle mass decline in individuals with DM, highlighting the impact of diabetes on physical function.

**Table 2:** Comparisons of sarcopenia-related parameters according to the diabetes mellitus status.

Sarcopenia-related parameters	Non-DM (n=66)		DM (n=64)		p
	lsm	se	lsm	se	
ASMI (kg/m^2^)	6.9	0.1	6.8	0.10	0.703
Body fat percentage (%)	32.7	0.66	32.4	0.67	0.751
Thigh circumference (cm)	49.7	0.37	49.3	0.37	0.464
Calf circumference (cm)	34.7	0.39	33.6	0.39	0.058
Handgrip Strength (kg) *	24.9	23.9–25.9	23.3	22.4–24.3	0.021
SPPB total score	10.9	0.20	9.9	0.21	0.001
5-time chair stand-up (sec) *	11.3	10.6–12.0	12.6	11.8–13.4	0.023
Timed up and go (TUG) (sec) *	11.1	10.6–11.6	12.4	11.8–13.0	0.001
4-meter gait speed (m/sec)	1.01	0.02	0.88	0.02	0.0002

*p-values are derived from the log-transformed data.

The analyses presented in this table were adjusted for age and sex.

ASMI, Appendicular skeletal muscle mass index; DM, diabetes mellitus; SPPB, short physical performance battery.

### 3.3 Comparisons of serum ApoJ and MSTN levels by sarcopenia status

**Figure 1:**
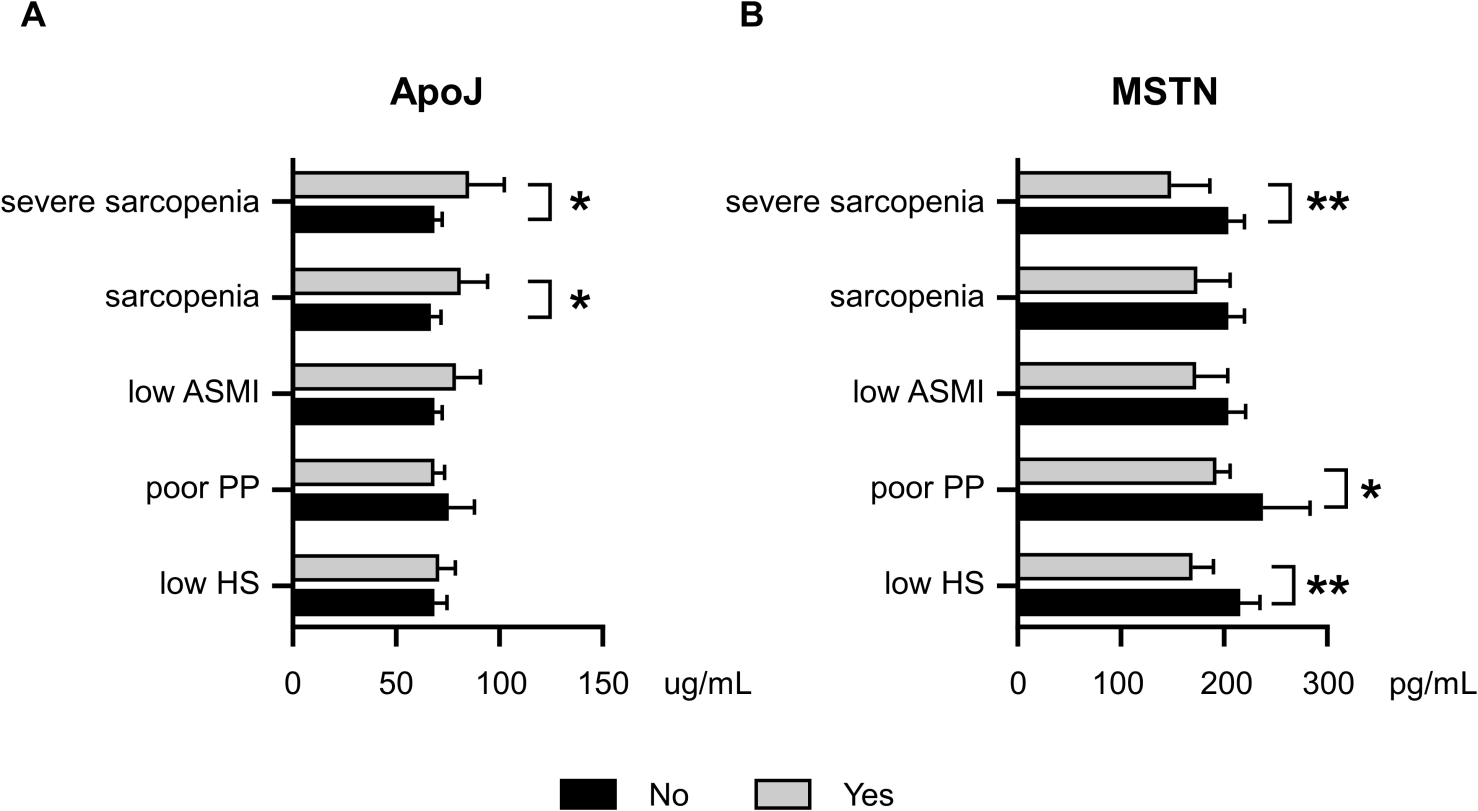
Age- and sex-adjusted serum ApoJ and MSTN levels according to Sarcopenia, severe Sarcopenia, and components of Sarcopenia. **(A)** ApoJ levels according to sarcopenia status and its components. **(B)** MSTN levels according to sarcopenia status and its components. * indicates a p-value of <0.05; ** indicates a p-value of <0.01 when comparing groups. ASMI, appendicular skeletal muscle mass index; ApoJ, apolipoprotein J; HS, handgrip strength; MSTN, myostatin; PP, physical performance.

**Figure 1** illustrates the age- and sex-adjusted serum ApoJ and MSTN levels in different groups according to sarcopenia status. Notably, significant differences were observed in the ApoJ levels between participants with and without sarcopenia [81.1 (69.9–94.1) μg/ml in the sarcopenia group vs. 66.9 (62.5–71.6) μg/ml in the no sarcopenia group, p=0.022] and severe sarcopenia [84.1 (68.9–102.6) μg/ml in the severe sarcopenia group vs. 67.7 (63.4–72.3) μg/ml in the non-severe sarcopenia group, p=0.044]. This finding indicates a potential association between higher ApoJ levels and the presence of sarcopenia (**Figure 1A**). However, individuals with poor PP or low HS did not show significant differences in ApoJ levels compared with their counterparts. ApoJ levels tended to increase in the low ASMI group (p=0.062). Conversely, the serum MSTN levels were significantly lower in participants with severe sarcopenia [149.1 (119.4–186.1) pg/ml vs. 204.0 (189.6–219.4) pg/ml, p=0.009], low HS [168.7 (150.0–189.8) pg/ml vs. 215.0 (197.4–234.0) pg/ml, p=0.001], or poor PP [190.9 (176.8–206.2) pg/ml vs. 236.9 (197.8–283.7) pg/ml, p=0.033] compared with their respective counterparts (**Figure 1B**). In addition, participants with low ASMI or sarcopenia tended to have lower MSTN levels (p=0.067 and p=0.098, respectively).

### 3.4 Associations of serum ApoJ and MSTN levels with sarcopenia and clinical covariates

In the age- and sex- adjusted Spearman’s correlation analysis (**Supplementary Table S2**), MSTN levels exhibited significant positive correlations with muscle mass and function, including ASMI (r = 0.211, p=0.017), HS (r = 0.274, p=0.002), and measures of PP, such as higher SPPB scores (r = 0.246, p=0.005) and shorter TUG times (r = −0.219, p=0.013). By contrast, the ApoJ levels showed no significant correlations with sarcopenia-related parameters or other clinical variables, except for a negative correlation with ALT (r = −0.197, p=0.026). No direct association was found between ApoJ and MSTN levels. Interestingly, when analyzing the correlation separately by DM status, a strong negative correlation between ApoJ levels and calf circumference was only observed in the DM group (r = −0.432, p=0.002). In participants with DM, the ApoJ levels had a significant correlation with poor PP, including the longer TUG times (r = 0.255, p=0.049) and slower 4-meter gait speed (r = −0.308, p=0.017) even after adjusting for muscle mass and physical activity (**Supplementary Table S3**). However, the difference in associations between ApoJ levels and sarcopenia-related parameters by DM status was found only for gait speed (p for interaction = 0.046, data not shown), with no interaction effects observed in other metrics.

With regard to MSTN (**Supplementary Table S4**), significant positive correlations were found between MSTN levels and key indicators of muscle mass, strength, and PP, including ASMI (r = 0.209, p = 0.018), HS (r = 0.222, p = 0.012), total SPPB scores (r = 0.243, p = 0.006), and TUG times (r = −0.225, p = 0.011). When analyzing the correlation between MSTN levels and clinical variables separately in the DM and non-DM groups, the significant correlation between MSTN levels and HS remained only in participants with DM. However, no significant interaction was found between MSTN levels and HS according to DM status (p for interaction = 0.82, data not shown). These findings suggest that MSTN levels are positively associated with muscle function across the study population, while ApoJ levels may have a distinct role in individuals with DM, particularly in relation to calf circumference and gait speed.

### 3.5 Logistic regression analysis of factors associated with sarcopenia and severe sarcopenia

Multiple logistic regression analyses were conducted to determine the impact of various factors, including ApoJ and MSTN levels, on the ORs for sarcopenia and severe sarcopenia (**Table 3**). Serum ApoJ level showed a significant positive association with both sarcopenia and severe sarcopenia after adjusting for confounding variables [OR: 1.027 (CI: 1.008–1.046) and OR 1.041 (CI: 1.005–1.079), respectively]. These findings suggest that higher levels of ApoJ are independently associated with an increased likelihood of sarcopenia. By contrast, serum MSTN level was inversely associated with severe sarcopenia [OR: 0.98 (CI: 0.964–0.997), p=0.025]. In a separate multiple logistic regression analysis evaluating the OR for low HS (data not shown), MSTN levels [OR: 0.994 (CI 0.988–1), p=0.034] were determined as an independent inverse association factor. In all logistic regression models, no significant interaction was observed based on the DM status (all p values for interaction > 0.3), suggesting the association of ApoJ and MSTN with sarcopenia-related parameters were independent of diabetes status.

**Table 3:** Logistic regression analysis of factors associated with Sarcopenia and severe Sarcopenia.

Variables	Sarcopenia			Severe Sarcopenia		
	OR	CI	p value	OR	CI	P-value
ApoJ	1.027	1.008–1.046	0.006	1.041	1.005–1.079	0.027
MSTN	0.993	0.987–1.002	0.120	0.98	0.964–0.997	0.025
Age	1.044	0.947–1.15	0.388	1.228	1.03–1.465	0.022
Men	3.457	1.013–11.80	0.048	1.665	0.24–11.572	0.606
BMI	0.708	0.576–0.87	0.001	0.73	0.55–0.969	0.029
DM	1.476	0.352–6.195	0.595	37.914	1.682–854.747	0.022
HTN	2.3	0.635–8.334	0.205	47.593	2.33–-972.029	0.012
Dyslipidemia	0.955	0.209–4.363	0.953	0.042	0.002–0.762	0.032
CVD	1.683	0.442–6.413	0.445	0.569	0.08–4.029	0.572
log GPAQ	0.65	0.443–0.954	0.028	0.541	0.314–0.93	0.026

ApoJ, apolipoprotein J; BMI, body mass index; CI, confidence interval; CVD, cardiovascular disease; DM, diabetes mellitus; HTN, hypertension; GPAQ, global physical activity questionnaire; OR, odds ratio; MSTN, myostatin.

### 3.6 Prevalence of sarcopenia according to serum ApoJ and MSTN levels

The participants were divided into four groups based on the median serum ApoJ and MSTN levels (**Table 4**). The High ApoJ/Low MSTN group had the highest prevalence of sarcopenia (26.7%, p=0.031) and severe sarcopenia (23.3%, p=0.024) compared with the other groups. In contrast, the Low ApoJ/High MSTN group had a prevalence of 0% for both sarcopenia or severe sarcopenia, suggesting a potential protective effect of high MSTN and low ApoJ levels against sarcopenia.

**Table 4:** Prevalence of Sarcopenia according to serum ApoJ and MSTN levels.

ApoJ/MSTN	Low/High (n=30)		Low/Low (n=35)		High/High (n=35)		High/Low (n=30)		P-value
	n	%	n	%	n	%	n	%	
Sarcopenia	0	0	7	20	8	22.9	8	26.7	0.009
Severe sarcopenia	0	0	3	8.6	3	8.6	7	23.3	0.022

ApoJ, apolipoprotein J; MSTN, myostatin.

Sarcopenia was defined as a low ASMI with either low handgrip strength or poor physical performance, while severe Sarcopenia was defined as a low ASMI with both low handgrip strength and poor physical performance.

## 4 Discussion

To the best of our knowledge, this study is the first to examine the association between circulating levels of ApoJ and sarcopenia in older adults with and without DM. We comprehensively assessed sarcopenia in older adults by evaluating muscle mass, muscle strength, and PP, while simultaneously measuring circulating levels of ApoJ and MSTN, which are representative hepatokines and myokines, respectively. Participants with sarcopenia or severe sarcopenia exhibited significantly higher levels of circulating ApoJ compared to those without sarcopenia. In contrast, MSTN levels were lower in individuals with reduced HS, impaired PP, or severe sarcopenia. These findings were supported by multivariable logistic regression analysis, which demonstrated that elevated ApoJ was independently associated with increased odds of both sarcopenia and severe sarcopenia. Conversely, higher MSTN levels were inversely associated with severe sarcopenia and low HS, independent of diabetes status. Additionally, older adults with DM exhibited notably lower MSTN levels and experienced a more pronounced decline in muscle function relative to muscle mass loss, suggesting that DM may predominantly impair muscle quality and functional capacity rather than merely reducing muscle mass.

### 4.1 Insulin resistance, diabetes and the potential role of ApoJ in sarcopenia

Numerous studies have suggested that IR and DM are contributors to the development of sarcopenia (4, 35). ApoJ has been implicated in skeletal muscle insulin signaling and may be associated with IR in humans (17, 18). Supporting this, Jeon et al. demonstrated that combined aerobic and resistance exercise training significantly reduced circulating ApoJ levels in patients with DM, and these changes were inversely associated with muscle mass gains and improvements in insulin sensitivity (36).

Our findings are generally consistent with prior observations, as we observed elevated ApoJ levels in individuals with sarcopenia. However, in our cohort, circulating ApoJ levels were not significantly correlated with HOMA-IR. This apparent discrepancy may be partially explained by the clinical characteristics of our study population, where over 78% of participants with DM had a disease duration exceeding 10 years. This prolonged disease course may have resulted in varying degrees of β-cell dysfunction, potentially confounding the expected associations with IR.

Given the complex interplay between hyperglycemia, IR, and impaired β-cell function in individuals with DM, we conducted Spearman correlation analyses stratified by DM status. Interestingly, a strong negative correlation between ApoJ levels, calf circumference, and gait speed was observed exclusively in participants with DM. Although the underlying mechanisms remain unclear, these findings raise the possibility that ApoJ may exert a more deleterious effect on muscle function in individuals with DM, warranting further investigation into its specific role in this population.

To further investigate whether the association between ApoJ and sarcopenia was mediated by metabolic dysfunction, we conducted additional multivariable logistic regression analyses adjusting for fasting plasma glucose, HbA1c, and HOMA-IR, independently of diabetes status. ApoJ remained a significant and independent correlate of sarcopenia in these models (data not shown). These results suggest that the observed relationship between ApoJ and sarcopenia is unlikely to be primarily mediated through IR or glucose metabolism. Instead, ApoJ may contribute to the development or progression of sarcopenia via alternative mechanisms, possibly through direct effects on muscle physiology or compensatory mechanisms. Further mechanistic studies are needed to elucidate these underlying mechanisms.

### 4.2 Potential mechanism linking ApoJ to sarcopenia

Although direct evidence linking ApoJ to skeletal muscle growth or differentiation remains limited, several studies have suggested associations between ApoJ and the disease activity of myositis (37), and others have reported that silencing ApoJ can help restore myoblast viability (20). Another plausible mechanism underlying our findings involves ApoJ’s function as a stress-induced molecular chaperone that protects various cell types from oxidative stress (19). This cytoprotective function is supported by studies showing elevated ApoJ expression in pathologic conditions characterized by increased oxidative stress, including coronary heart disease and several types of cancer (38–40). Therefore, it is conceivable that elevated circulating ApoJ levels observed in individuals with sarcopenia may reflect a compensatory response to cellular oxidative stress in skeletal muscle. Further studies should aim to elucidate the mechanistic role of ApoJ in muscle cell differentiation, oxidative damage response, and muscle-liver communication, as these pathways may reveal new therapeutic targets or biomarkers for sarcopenia.

### 4.3 The role of MSTN in muscle function and sarcopenia

MSTN has garnered attention as a key myokine in the regulation of muscle homeostasis, particularly after observations that MSTN knockout in mice resulted in marked muscle hypertrophy (41, 42). Despite these preclinical findings, clinical trials evaluating MSTN antibodies as potential therapies for sarcopenia have thus far failed to demonstrate clinically meaningful benefits (42). In humans, studies examining the relationship between serum MSTN levels and muscle strength have yielded inconsistent results, possibly due to heterogeneity in study populations, comorbid conditions, or the influence of confounding interventions such as exercise. In our cohort of community-dwelling older adults with and without DM, higher circulating MSTN levels were positively associated with better muscle strength and enhanced PP, even after adjusting for muscle mass and physical activity. This association remained robust in ANCOVA analyses comparing MSTN levels between participants with and without low HS (data not shown), with lower MSTN levels consistently observed among those with low HS even after further adjusting for age, sex, and related variables such as SBP, HOMA-β, total cholesterol, and creatine phosphokinase levels. Supporting these findings, a recent study in patients with chronic kidney disease reported elevated MSTN levels following 12 months of exercise training (43), suggesting a potential link between MSTN and muscle adaptation to physical activity. Furthermore, MSTN-knockout mouse models, while demonstrating muscle hypertrophy, exhibited compromised force generation and were associated with reduced oxidative capacity in skeletal muscle fibers (44). Collectively, these findings suggest that MSTN may serve not merely a negative regulator of muscle mass, but also as a potential biomarker of muscle function and quality in older adults with age-related sarcopenia.

### 4.4 Study limitations

Several limitations should be carefully considered when interpreting the findings. First, due to its cross-sectional design, causality between ApoJ and MSTN levels and sarcopenia cannot be established. To address this limitation, we are currently conducting 1-year longitudinal follow-up within the same cohort, which is expected to provide more robust temporal data to clarify the directionality of these associations. Second, this study was conducted within a single ethnic population and included a relatively small sample size, which may limit the generalizability of the findings. As an exploratory study, our aim was to investigate associations between circulating biomarkers and sarcopenia-related parameters. Given the limited research on whether the effects of ApoJ and MSTN on muscle physiology differ by race or ethnicity, further multi-ethnic studies should be conducted to explore potential variations. To reduce potential bias associated with the small number of events in our regression models, logistic regression analyses were additionally performed using Firth’s penalized maximum likelihood estimation (**Supplementary Table S5**). Lastly, although we adjusted for potential confounding factors, the influence of residual confounding, including unmeasured lifestyle factors or the effects of medications on muscle mass and function, cannot be entirely ruled out. Future studies with larger, more diverse populations and longitudinal designs are necessary to validate and expand upon our findings.

### 4.5 Strengths and implications of the study

Despite the aforementioned limitations, this study represents the first to concurrently assess circulating levels of the hepatokine ApoJ and the myokine MSTN alongside muscle mass, muscle strength, and PP—the three core diagnostic domains of sarcopenia—to comprehensively examine their associations with sarcopenia in older adults. To enhance the robustness of our analyses and minimize the impact of potential confounders, we also conducted detailed nutritional assessments and administered a validated physical activity questionnaire. These complementary evaluations allowed for a more thorough characterization of factors influencing muscle quantity and function. Importantly, our novel findings may help bridge existing knowledge gaps, particularly in understanding hepatokine-myokine interactions and their role in liver-muscle communication. This insight could guide future investigations into ApoJ and MSTN as potential biomarkers and interventions for preventing sarcopenia in older adults.

### 4.6 Conclusion

Elevated circulating ApoJ levels were independently associated with an increased risk of sarcopenia, whereas higher MSTN levels were positively correlated with greater muscle strength and better PP in older adults. These findings suggest that ApoJ and MSTN may serve as potential biomarkers, each reflecting distinct aspects of muscle physiology relevant to sarcopenia. However, further longitudinal studies and mechanistic research are warranted to validate these associations and to elucidate the roles of ApoJ and MSTN in muscle physiology and sarcopenia progression.

## Data Availability

The datasets presented in this article are not readily available because further inquiries can be directed to the corresponding author. Requests to access the datasets should be directed to seojia@korea.ac.kr.

## Glossary

Appendicular Skeletal Muscle Mass (ASM): The sum of lean muscle mass in the upper and lower extremities, measured using dual-energy X-ray absorptiometry (DXA)
Appendicular Skeletal Muscle Mass Index (ASMI): A measure of muscle mass calculated by dividing appendicular skeletal muscle mass (ASM) by height in meters squared (kg/m²). Low ASMI is defined as <7.0 kg/m²
Short Physical Performance Battery (SPPB): A series of physical tests used to assess lower extremity function, including balance, 4-meter gait speed, and 5-time chair stand-up test. Scores range from 0 to 12, with higher scores indicating better physical performance. Individuals with an SPPB score of ≤9 were considered to have poor PP
Timed Up and Go (TUG) Test: A functional mobility test used in sarcopenia assessments. It measures the time taken to rise from a chair, walk 3 meters, turn around, walk back, and sit down. According to the Korean Working Group on Sarcopenia (KWGS) guideline, a TUG time of ≥12 seconds is considered indicative of poor physical performance
Sarcopenia: Based on the clinical guidelines of the KWGS, defined as decreased Appendicular Skeletal Muscle Mass Index (ASMI) combined with either low muscle strength or poor physical performance (PP)
Severe Sarcopenia: Based on the clinical guidelines of the KWGS, defined as decreased ASMI along with both low muscle strength and poor physical performance
