# Supplementary material for: Associations of circulating apolipoprotein J and myostatin with sarcopenia in older adults with and without type 2 diabetes: a cross-sectional study
Source: Front Endocrinol (Lausanne). 2025 Jun 30;16:1592112. doi: 10.3389/fendo.2025.1592112 (PMC12256245; doi:10.3389/fendo.2025.1592112)
Supplement: Supplementary file 1 [file DataSheet1.docx]

**Supplementary Methods: Operational Definition and Assessment of Sarcopenia**

**1. Appendicular Skeletal Muscle Mass Index (ASMI)**

Appendicular skeletal muscle mass (ASM) was measured using dual-energy X-ray absorptiometry (DEXA; Lunar Radiation, Madison, WI, USA). Participants were asked to lie in a supine position on the DEXA table, wearing light clothing and no metallic objects. Scans were performed by trained technicians according to standardized protocols. The ASM was calculated as the sum of lean soft tissue mass in both the upper and lower limbs. The ASMI was then derived by dividing the ASM by the square of the participant’s height in meters (kg/m²). Low muscle mass was defined as an ASMI of <7.0 kg/m² in men and <5.4 kg/m² in women, consistent with the cut-off values recommended by the Asian Working Group for Sarcopenia (Chen et al., 2020).

**2. Muscle Strength - Handgrip Strength (HS)**

Muscle strength was assessed using a digital hand dynamometer (TKK-5401; Takei®, Tokyo, Japan). Participants were instructed to stand upright with feet shoulder-width apart. Each participant held the dynamometer in their dominant hand, fully extended the arm without elbow flexion, and was encouraged to squeeze the device as forcefully as possible. Two trials were conducted for each hand, and the maximum value obtained from all four attempts was recorded as the final handgrip strength (kg). The measurements were taken under the supervision of trained personnel. Low muscle strength was defined as <28 kg for men and <18 kg for women.

**3. Physical Performance (PP)**

Physical performance was evaluated using the Short Physical Performance Battery (SPPB), which includes three components: balance, gait speed, and chair stand. The balance test included side-by-side, semi-tandem, and full tandem stances held for up to 10 seconds each. Gait speed was measured over a 4-meter straight path marked with tape, with participants instructed to walk at their usual pace. Time was measured using a stopwatch, and the average speed in meters per second was calculated. For the chair stand test, participants were asked to fold their arms across their chest and stand up and sit down from a standard-height chair (43 cm) five times as quickly as possible without using their arms.

Additionally, performance was cross-validated using a multi-sensor-based kiosk system (AndanteFit, Dyphi, Daejeon, South Korea), which automatically recorded gait parameters and chair-stand timings using pressure and motion sensors. Timed Up and Go (TUG) testing was also performed, where participants stood up from a chair, walked 3 meters, turned around, walked back, and sat down again. Poor physical performance was defined as any of the following: SPPB score ≤9, chair stand test >10 seconds, 4-meter gait speed <1.0 m/s, or TUG test ≥12 seconds.

**4. Definition of Sarcopenia**

Sarcopenia was diagnosed when participants had a low ASMI in combination with either low HS or poor PP. Severe sarcopenia was diagnosed when all three abnormalities (low ASMI, low HS, and poor PP) were present.
