# Supplementary material for: Associations of circulating apolipoprotein J and myostatin with sarcopenia in older adults with and without type 2 diabetes: a cross-sectional study
Source: Front Endocrinol (Lausanne). 2025 Jun 30;16:1592112. doi: 10.3389/fendo.2025.1592112 (PMC12256245; doi:10.3389/fendo.2025.1592112)
Supplement: Supplementary file 2 [file DataSheet2.docx]

**Supplementary Table S1. Characteristics of the study participants**

|  | **Non-DM** | | | **DM** | | |  | |  |
| --- | --- | --- | --- | --- | --- | --- | --- | --- | --- |
|  |  |  | |  |  | | p-value | |  |
| Calcium* (mg/dL) | 9.4 | 9.2–9.7 | | 9.5 | 9.3–9.7 | | 0.048 | |  |
| Phosphorus (mg/dL) | 3.4 | 0.44 | | 3.6 | 0.53 | | 0.036 | |  |
| 25-OH-Vit D3 (ng/mL) | 30.7 | 11.9 | | 27.6 | 11.7 | | 0.142 | |  |
| Total cholesterol* (mg/dL) | 188 | 141−210 | | 134 | 118−156 | | <.0001 | |  |
| HDL cholesterol (mg/dL) | 57.1 | 12.7 | | 51.2 | 11.8 | | 0.007 | |  |
| LDL cholesterol* (mg/dL) | 112 | 78−134 | | 67.5 | 52.5–86.5 | | <.0001 | |  |
| Triglyceride* (mg/dL) | 96.5 | 77−134 | | 116.0 | 86–153.5 | | 0.105 | |  |
| hs-CRP* (mg/dL) | 0.07 | 0.04−0.13 | | 0.05 | 0.03−0.13 | | 0.180 | |  |
| Urine ACR* (mg/g) | N/A | | | 22.1 | 10.2–51.2 | |  | |  |
| Microvascular complication (n,%) | N/A | | | 35 | | 54.7 |  | |  |
| Duration of diabetes (n, %) |  | |  |  | |  |  | |  |
| <5 yr |  | |  | 10 | | 15.6 |  | |  |
| 5–9 yr |  | |  | 4 | | 6.3 |  | |  |
| 10–14 yr |  | |  | 7 | | 10.9 |  | |  |
| ≥15 yr |  | |  | 43 | | 67.2 |  | |  |
| Low ASMI (n, %) | 10 | | 15.2 | 14 | | 21.9 | | 0.323 | |
| Low handgrip strength (n, %) | 16 | | 24.2 | 29 | | 45.31 | | 0.012 | |
| Poor physical performance (n, %) | 52 | | 78.8 | 57 | | 89.1 | | 0.112 | |
| SPPB ≤9 | 13 | | 19.7 | 22 | | 34.38 | |  | |
| 5-time chair stand-up >10 sec | 47 | | 71.21 | 52 | | 81.25 | |  | |
| 4-m gait speed <1.0 m/sec | 33 | | 50 | 44 | | 68.75 | |  | |
| Timed up and go ≥12 sec | 23 | | 34.85 | 34 | | 53.13 | |  | |

Abbreviations: ACR, albumin-to-creatinine ratio; ASMI, appendicular skeletal muscle mass index; DM, diabetes mellitus; HDL, high-density lipoprotein; hs-CRP, high-sensitivity C-reactive protein; LDL, low-density lipoprotein; SPPB, short physical performance battery

* Variables that did not meet the assumption of normality, as assessed by the Shapiro–Wilk test and Q–Q plot, are presented as median (interquartile range). These include calcium, total cholesterol, LDL cholesterol, triglyceride, hs-CRP, and urine ACR. Skewed variables were log-transformed for statistical analysis where applicable.

**Supplementary Table S2. Spearman’s correlation between age- and sex-adjusted ApoJ/MSTN levels and clinical variables**

|  |  | | **Age- and sex-adjusted ApoJ** | | **Age- and sex-adjusted MSTN** | |
| --- | --- | --- | --- | --- | --- | --- |
|  | | **n** | **r** | ***P*** | **r** | ***P*** |
| BMI (kg/m^2^) | | 130 | 0.039 | 0.659 | 0.035 | 0.693 |
| Waist circumference (cm) | | 130 | –0.013 | 0.881 | 0.007 | 0.937 |
| SBP (mmHg) | | 130 | –0.057 | 0.523 | 0.271 | 0.002 |
| FPG (mg/dL) | | 130 | 0.014 | 0.872 | –0.069 | 0.438 |
| HbA1c (%) | | 130 | –0.018 | 0.839 | –0.165 | 0.062 |
| Fasting insulin (μU/mL) | | 129 | –0.009 | 0.919 | 0.143 | 0.110 |
| HOMA-IR* | | 129 | 0.000 | 0.997 | 0.115 | 0.199 |
| HOMA-β | | 129 | –0.015 | 0.863 | 0.212 | 0.017 |
| Total cholesterol (mg/dL) | | 130 | 0.095 | 0.289 | 0.203 | 0.022 |
| HDL cholesterol (mg/dL) | | 130 | 0.079 | 0.373 | 0.126 | 0.157 |
| LDL cholesterol (mg/dL) | | 130 | 0.033 | 0.712 | 0.197 | 0.026 |
| Triglyceride* (mg/dL) | | 130 | 0.130 | 0.144 | –0.018 | 0.844 |
| hs-CRP (mg/dL)* | | 129 | –0.022 | 0.804 | –0.068 | 0.451 |
| ALT* (IU/L) | | 130 | –0.197 | 0.026 | 0.036 | 0.690 |
| AST* (IU/L) | | 130 | –0.082 | 0.359 | 0.165 | 0.062 |
| CPK (IU/L) | | 130 | –0.033 | 0.713 | 0.279 | 0.001 |
| BUN (mg/dL) | | 130 | –0.082 | 0.358 | –0.113 | 0.206 |
| Serum creatinine (mg/dL) | | 130 | 0.024 | 0.789 | 0.109 | 0.220 |
| 25-OH-Vit D3 (ng/mL) | | 130 | –0.114 | 0.200 | 0.029 | 0.749 |
| Calcium (mg/dL) | | 130 | –0.012 | 0.893 | –0.085 | 0.341 |
| Phosphorus (mg/dL) | | 130 | –0.028 | 0.753 | –0.105 | 0.238 |
| ApoJ (µg/mL) | | 130 | NA | NA | –0.035 | 0.6977 |
| Thigh circumference (cm) | | 130 | 0.013 | 0.881 | 0.093 | 0.295 |
| Calf circumference (cm) | | 101 | –0.138 | 0.173 | 0.116 | 0.252 |
| ASMI (kg/m^2^) | | 130 | –0.097 | 0.275 | 0.211 | 0.017 |
| Total lean muscle (kg) | | 130 | –0.002 | 0.983 | 0.107 | 0.227 |
| Body fat percentage (%) | | 130 | 0.052 | 0.560 | –0.145 | 0.103 |
| Handgrip Strength (kg) | | 130 | 0.003 | 0.972 | 0.274 | 0.002 |
| Timed Up and Go (TUG) (s) | | 130 | 0.062 | 0.490 | –0.219 | 0.013 |
| 4-m gait speed (m/s) | | 130 | –0.023 | 0.794 | 0.160 | 0.071 |
| 5-time chair stand-up (s) | | 130 | –0.005 | 0.956 | –0.178 | 0.044 |
| SPPB total score | | 130 | –0.021 | 0.814 | 0.246 | 0.005 |
| GPAQ (METs/week) | | 130 | –0.039 | 0.665 | 0.031 | 0.730 |

Abbreviation: ALT, alanine aminotransferase; ApoJ, apolipoprotein J; ASMI, appendicular skeletal muscle mass index; AST, aspartate aminotransferase; BMI, body mass index; BUN, blood urea nitrogen; CPK, creatinine phosphokinase; CVD, cardiovascular disease; DM, diabetes mellitus; FPG, fasting plasma glucose; GPAQ, global physical activity questionnaire; HbA1c, hemoglobin A1c; HDL, high-density lipoprotein; HOMA-IR, homeostasis model assessment of insulin resistance; HOMA-β, homeostasis model assessment of β-cell function; hs-CRP, high-sensitivity C-reactive protein; LDL, low-density lipoprotein; METs, metabolic equivalents; MSTN, myostatin; NA, not available; SBP, systolic blood pressure; SPPB, short physical performance battery

* Non-normally distributed variables were analyzed after logarithmic transformation.

**Supplementary Table S3. Partial Spearman’s correlation between ApoJ and handgrip strength and physical performance**

|  | **All** | | | | **non-DM** | | | **DM** | | |
| --- | --- | --- | --- | --- | --- | --- | --- | --- | --- | --- |
|  | | **n** | **r** | ***P*** | **n** | **r** | ***P*** | **n** | **r** | ***P*** |
| Handgrip strength (kg) | | 130 | 0.040 | 0.659 | 66 | 0.156 | 0.227 | 64 | −0.039 | 0.768 |
| Timed Up and Go (TUG) (s) | | 130 | 0.056 | 0.535 | 66 | −0.112 | 0.387 | 64 | 0.255 | 0.049 |
| 4-m gait speed (m/s) | | 130 | −0.018 | 0.845 | 66 | 0.210 | 0.101 | 64 | −0.308 | 0.017 |
| 5-time chair stand-up (s) | | 130 | −0.011 | 0.905 | 66 | −0.120 | 0.353 | 64 | 0.086 | 0.51 |
| SPPB total score | | 130 | −0.014 | 0.879 | 66 | 0.145 | 0.262 | 64 | −0.230 | 0.077 |
| ASMI* | | 130 | −0.093 | 0.297 | 66 | 0.030 | 0.816 | 64 | −0.189 | 0.144 |

Adjusted for age, sex, ASMI, and GPAQ scores

*Adjusted for age, sex, and GPAQ scores

Abbreviations: ASMI, appendicular skeletal muscle mass index; GPAQ, Global Physical Activity Questionnaire; SPPB, Short Physical Performance Battery

**Supplementary Table S4. Partial Spearman’s correlation of myostatin with handgrip strength and physical performance**

|  | **All** | | | **non-DM** | | | | **DM** | | |
| --- | --- | --- | --- | --- | --- | --- | --- | --- | --- | --- |
|  | **n** | **r** | ***P*** | | **n** | **r** | ***P*** | **n** | **r** | ***P*** |
| Handgrip strength | 130 | 0.222 | 0.012 | | 66 | 0.055 | 0.671 | 64 | 0.315 | 0.014 |
| Timed Up and Go (TUG) (s) | 130 | −0.225 | 0.011 | | 66 | −0.189 | 0.141 | 64 | −0.109 | 0.408 |
| 4-m gait speed (m/s) | 130 | 0.167 | 0.062 | | 66 | 0.141 | 0.275 | 64 | 0.064 | 0.625 |
| 5-time chair stand-up (s) | 130 | −0.171 | 0.055 | | 66 | −0.116 | 0.370 | 64 | −0.185 | 0.157 |
| SPPB total score | 130 | 0.243 | 0.006 | | 66 | 0.205 | 0.110 | 64 | 0.212 | 0.104 |
| ASMI* | 130 | 0.209 | 0.018 | | 66 | 0.169 | 0.185 | 64 | 0.146 | 0.261 |

Adjusted for age, sex, ASMI and GPAQ scores

*Adjusted for age, sex, and GPAQ scores

Abbreviations: ASMI, appendicular skeletal muscle mass index; GPAQ, Global Physical Activity Questionnaire; SPPB, Short Physical Performance Battery

**Supplementary Table S5. Multivariable logistic regression analysis of factors associated with sarcopenia and severe sarcopenia using Firth’s penalized likelihood method**

|  | **Sarcopenia** | | | **Severe Sarcopenia** | | |
| --- | --- | --- | --- | --- | --- | --- |
|  | OR | CI | p value | OR | CI | p-value |
| ApoJ | 1.023 | 1.005–1.04 | 0.011 | 1.03 | 1.003–1.057 | 0.029 |
| MSTN | 0.995 | 0.987–1.002 | 0.158 | 0.986 | 0.973–0.999 | 0.035 |
| Age | 1.039 | 0.933–1.137 | 0.404 | 1.157 | 1.013–1.323 | 0.032 |
| Men | 2.863 | 0.933–8.785 | 0.066 | 1.403 | 0.292–6.739 | 0.672 |
| BMI | 0.75 | 0.624–0.901 | 0.002 | 0.801 | 0.644–0.996 | 0.046 |
| DM | 1.386 | 0.379–5.067 | 0.622 | 11.449 | 1.292–101.472 | 0.029 |
| HTN | 2.063 | 0.635–6.706 | 0.229 | 13.819 | 1.676–-113.965 | 0.015 |
| Dyslipidemia | 0.947 | 0.241–3.729 | 0.938 | 0.111 | 0.014–0.902 | 0.040 |
| CVD | 1.604 | 0.466–5.524 | 0.454 | 0.662 | 0.126–3.471 | 0.626 |
| log GPAQ | 0.677 | 0.479–0.959 | 0.028 | 0.632 | 0.42–0.95 | 0.028 |

Logistic regression analyses were performed using Firth’s penalized maximum likelihood estimation to reduce bias due to small event numbers.

Abbreviations: ApoJ, apolipoprotein J; BMI, body mass index; CI, confidence interval; CVD, cardiovascular disease; DM, diabetes mellitus; HTN, hypertension; GPAQ, global physical activity questionnaire; OR, odds ratio; MSTN, myostatin
